# Recent Advances in the Synthesis of High Boron-Loaded Nucleic Acids for BNCT

**DOI:** 10.3389/fchem.2021.619052

**Published:** 2021-03-15

**Authors:** Darya Sergeevna Novopashina, Mariya Alexandrovna Vorobyeva, Alya Venyaminova

**Affiliations:** Laboratory of RNA Chemistry, Institute of Chemical Biology and Fundamental Medicine SB RAS, Novosibirsk, Russia

**Keywords:** boron cluster, oligonucleotide conjugate, conjugation method, pre- and post-synthetic modification, BNCT, boron neutron capture therapy

## Abstract

Boron clusters attract considerable attention as promising therapeutic tools for boron neutron capture therapy (BNCT). They combine high boron content with high chemical and biological stability, biorthogonality, and low toxicity. The development of oligonucleotide-based constructs and nucleic acid-like molecules, such as oligomeric phosphate diesters, bearing one or multiple boron clusters permits to create potential high boron-loaded agents for BNCT with good bioavailability, specifically interacting with nucleic acids inside the cell. Here, we shortly review the strategies and solutions in the design of oligonucleotide conjugates with boron clusters in light of the requirements for effective BNCT and future prospects of their practical use.

## Introduction

Boron neutron capture therapy (BNCT) is a binary chemoradiotherapeutic approach to cancer treatment that employs boron-containing drugs for the selective killing of tumor cells by the irradiation of low-energy neutrons (epithermal neutrons) (see, e.g., Calabrese et al., 2018). Sufficient application of this method in clinical medicine requires, first of all, effective and selective accumulation of boron compounds in the cancer cells. The creation of novel sources of epithermal neutrons with improved characteristics together with molecular engineering of new boron delivery agents can lead to the breakthrough in the practical use of the BNCT for the therapy of the tumors, especially those resistant to other treatments (e.g., Taskaev, 2015; Barth et al., 2018; Sato et al., 2018; Taskaev, 2019; Dymova et al., 2020).

The most important requirements to boron-containing agents for BNCT include low toxicity, high efficiency and selectivity of intra-tumor delivery (approx. 20–35 *μ*g/g, or 10^9 10^B atoms/cell), sufficient level of epithermal neutrons capture to produce a localized cell-destroying nuclear reaction, residence in the tumor during some hours and, respectively, fast enough clearance from normal tissues and blood (Soloway et al., 1998; Hawthorne and Lee, 2003; Sivaev and Bregadze, 2009). To the moment, only two low molecular weight boron drugs have been approved for clinical use: boron phenylalanine and sodium borocaptate (see, e.g., Ali et al., 2020). However, both of them hardly meet the criteria mentioned above. An intensive search is still ongoing for possibilities of maximal loading of BNCT agents by the boron-10. From this viewpoint, polyhedral boron clusters attract particular attention as the molecular moieties of choice for the design of ^10^B-delivery agents (Leśnikowski, 2020). The synthesis, characterization, and properties of polyhedral boranes and their derivatives have been the topic of several comprehensive monographs (see, e.g., Olah et al., 1991; Davidson et al., 2007; Grimes, 2016; Hey-Hawkins and Teixidor, 2018) and review articles (see, e.g., Sivaev et al., 2002; Kanazawa et al., 2019).

Conjugation of *closo*-dodecaborate and carboranes with different biomolecules (amino acids, peptides, nucleic acid bases, nucleosides, DNA binding molecules, carbohydrates, porphyrins), known drugs, and other tumor-targeting compounds provides a vast diversity of potential low molecular weight boron delivery agents with improved uptake and favorable pharmacokinetic characteristics (Druzina et al., 2016; Barth et al., 2018; Ignatova et al., 2020; Tsurubuchi et al., 2020 and references therein). Monoclonal antibodies, liposomes, polymers, dendrimers, and different types of nanoparticles have been intensively studied as targeted high molecular weight carriers for boron clusters due to the possibility to load them with high ^10^B content and to functionalize the surface of constructs with additional tumor-targeted ligands (Wu et al., 2006; Calabrese et al., 2012; Laurentia and Rodica, 2016; Barth et al., 2018; Hu et al., 2020 and references therein).

The ability of synthetic oligonucleotides of specific interaction with the genetic material (DNA and RNA) within cytoplasm or nucleus makes them a very promising platform to construct effective boron carriers for BNCT. Simultaneously, boron clusters represent attractive modifiers for nucleic acids due to their high boron content, relative metabolic inertness, low toxicity, and multiple possibilities to incorporate them into nucleic acids.

Joining nucleic acid and boron chemistries opens wide opportunities to create new effective boron-rich agents for BNCT. This issue is illustrated, in particular, in the series of publications by V. Bregadze’s team on the synthesis and investigation of nucleic acids precursors modified by polyhedral boron clusters (Sivaev et al., 2000; Semioshkin et al., 2007; Sivaev and Bregadze, 2009; Druzina et al., 2016 and others), as well as by a large cycle of publications by Lesnikowski with co-workers on developing synthetic methods for incorporation of different types of boron clusters into nucleosides and oligonucleotides (see reviews and some papers, e.g., Schinazi et al., 1994; Schinazi and Lesnikowski, 1998; Lesnikowski et al., 1999; Olejniczak et al., 2002; Lesnikowski, 2003; Kwiatkowska et al., 2013; Olejniczak et al., 2013; Kaniowski et al., 2017; Olejniczak et al., 2018). The progress in the development of modular and versatile synthetic approaches to the ^10^B-modification of oligonucleotides and the right balance of the properties of two constituents of the construct substantially extended the field of application for oligonucleotide-boron conjugates. They are considered now as perspective high loaded boron carriers for BNCT, antisense/antigene therapeutic agents and tools for molecular diagnostics, electrochemical biosensors, IR-sensitive probes, and modules for nanoconstructs (Schinazi et al., 1994; Lesnikowski, 2003; Olejniczak et al., 2005; ; Lesnikowski, 2007; Ziółkowski et al., 2012; Kwiatkowska et al., 2013; Olejniczak et al., 2013; Kaniowski et al., 2017; Olejniczak et al., 2018; Kaniowski et al., 2020a).

In this mini-review, we make a particular focus on the progress in the synthesis of different types of oligonucleotide-based constructs, including antisense DNA oligonucleotides, siRNA, and oligomeric phosphate diesters bearing one or multiple boron clusters in light of the requirements for effective BNCT and future trends in the design of functional nucleic acids and their composites as new potent BNCT agents.

## Approaches for the introduction of boron clusters into oligonucleotides and oligophosphates

Different synthetic methods have been developed to incorporate boron clusters into oligonucleotides and oligophosphates. These approaches include solid-phase phosphoramidite, H-phosphonate, and triester methods, as well as post-synthetic modifications. Typically, the conjugates are characterized by standard methods, such as reverse-phase high-performance liquid chromatography (RP HPLC), polyacrylamide gel electrophoresis (PAGE), mass-spectrometry (ESI-MS or MALDI-TOF MS), CD-spectroscopy, and thermal denaturation of duplexes. IR-spectroscopy can also be used for B-H signal determination (Kaniowski et al., 2017). However, this method has limited usage due to the low intensity of the B-H signal compared to other IR-signals from oligonucleotide. Therefore, IR-spectroscopy suits only for relatively short oligonucleotides or multiply boron-modified oligonucleotide conjugates. Determination of hydrophobicity characteristics (log*P* or log*D* values) also makes use for estimating the hydrophobicity of oligonucleotide-boron cluster conjugates (Kwiatkowska et al., 2013; Ebenryter-Olbińska et al., 2017).

### Types of Boron Clusters

**FIGURE 1:**
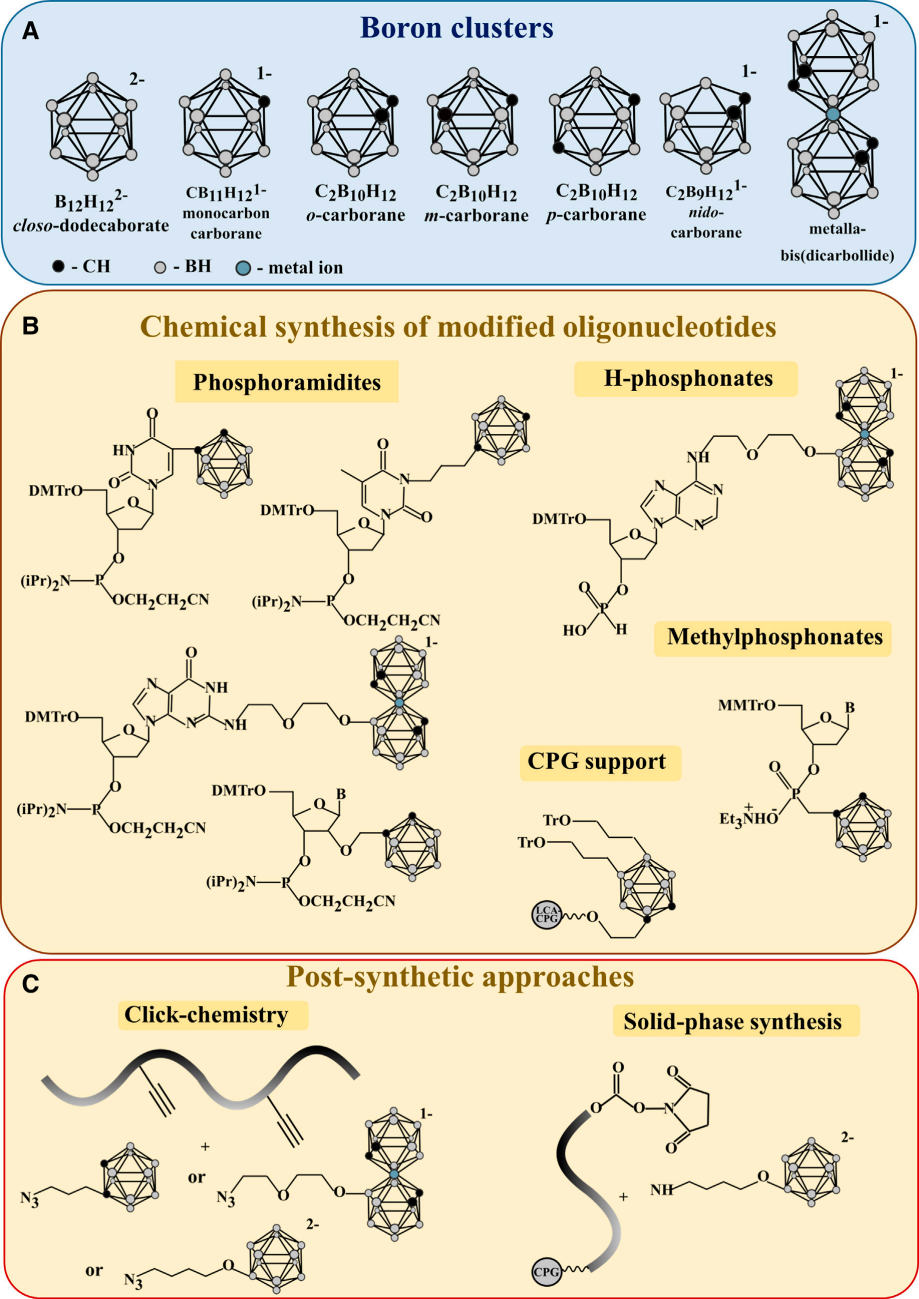
**(A)** Boron clusters applied in the engineering of biologically active boron-modified compounds **(B)** Monomeric synthons and polymer support for the solid-phase synthesis of oligonucleotides bearing boron clusters **(C)** Post-synthetic approaches for the incorporation of boron clusters into oligonucleotides.

A large repertoire of boron clusters is available for the creation of biologically active structures (see, e.g., Lesnikowski, 2011). Figure 1A shows negatively charged *closo*-dodecaborates (B_12_H_12_^2-^) and monocarbon carboranes (C_1_B_10_H_12_^1-^), electroneutral carboranes (*ortho*-, *meta*- and *para*- C_2_B_10_H_12_), their open form *nido*-carboranes (C_2_B_9_H_12_^1-^), and metallacarboranes.

Boron clusters can be converted to phosphoramidite, H-phosphonate, or methylphosphonate monomeric synthons (see, e.g., Lesnikowski, 2003; Olejniczak et al., 2018) or to specially modified polymer support (Janczak et al., 2015; Kaniowski et al., 2020a) for the incorporation during the solid-phase synthesis of oligonucleotides (Figure 1B). As an alternative post-synthetic approach, amino derivatives of boron clusters can be attached to activated 5′-hydroxyl of polymer-bound oligonucleotides (Meschaninova et al., 2019). Also, azido-modified boron clusters can react with alkyne-bearing oligonucleotides *via* "click" chemistry in solution (Ebenryter-Olbińska et al., 2017; Kaniowski et al., 2017) (Figure 1C).

The synthesis of nucleoside monomers bearing boron clusters is usually multi-stage, laborious, and time-consuming. More convenient ways for introducing boron clusters into nucleosides were proposed recently, based on cross-coupling and [3 + 2]-bipolar cycloaddition (see, e.g., the detailed review of Druzina et al., 2016 and references therein). Boron-modified nucleoside triphosphates, which serve as substrates for DNA polymerases, offer another possibility to incorporate boron clusters into DNA (Balintová et al., 2017).

### Solid-phase Approaches

**FIGURE 2:**
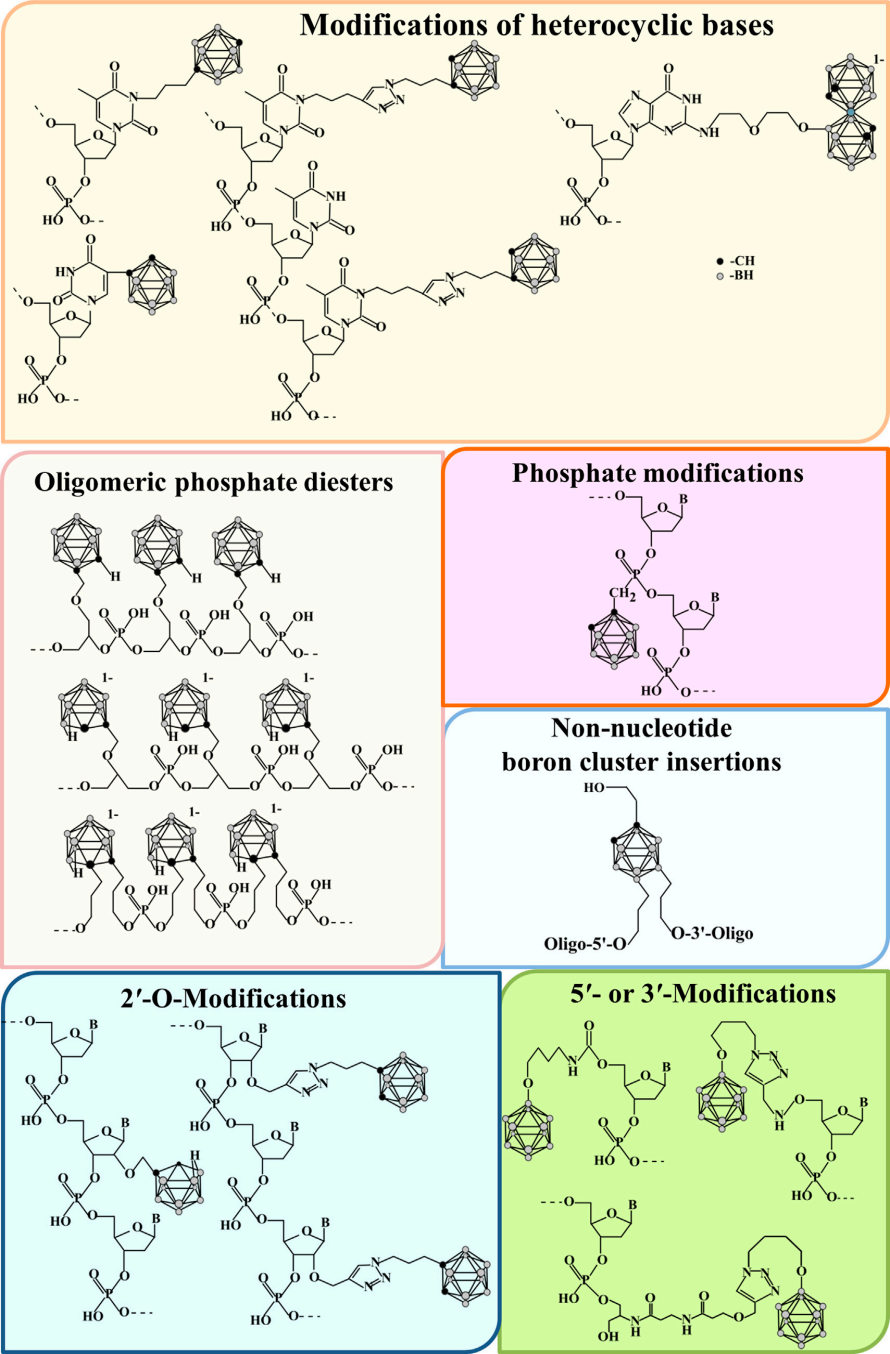
The examples of conjugates of nucleic acids and NA-like oligomeric phosphate diesters with boron clusters.

Solid-phase H-phosphonate and phosphoramidite methods are used for the synthesis of oligonucleotides modified by boron clusters (Figure 1B and Figure 2). Incorporation of boron clusters into heterocyclic bases or 2′-position of the ribose ring is performed by phosphoramidite method using cognate modified nucleoside phosphoramidites (Kwiatkowska et al., 2013; Matuszewski et al., 2015; Olejniczak et al., 2018). Metallacarboranes are introduced by a combination of phosphoramidite and H-phosphonate methods using modified nucleoside H-phosphonates (Olejniczak et al., 2007; Olejniczak, 2011; Ziółkowski et al., 2012; Olejniczak et al., 2018). To introduce boron clusters to the internucleoside phosphodiester linkage, one should use the triester method and methylphosphonates (Lesnikowski et al., 1999). An original approach to the synthesis of carborane-modified oligonucleotides developed by Lesnikowski and Nawrot with co-authors (Janczak et al., 2015; Kaniowski et al., 2020a) allows for solid-phase synthesis of so-called "oligopeds," carboranes multifunctionalized by several short DNAs with similar structure (e.g., antisense oligonucleotides) as future building blocks for nanoconstructs. Of note, deblocking of carborane-modified oligonucleotides in basic conditions gives a mixture of *closo*- and *nido*-derivatives, which can be separated by the reverse-phase HPLC (Lesnikowski, 2003; Matuszewski et al., 2015).

An interesting class of high boron-loaded carriers was proposed on the basis of oligomeric phosphate diesters with a regular NA-like backbone, in which acyclic diol replaces the sugar moiety, and carborane cages stand instead of nucleobases (Kane et al., 1993a; Kane et al., 1993b; Chen et al., 1994; Drechsel et al., 1994; Kane et al., 1996; Guan et al., 1998; Nakanishi et al., 1999; Lesnikowski et al., 2004) (Figure 2). In common with oligonucleotides, oligophosphates are produced mainly by a solid-phase phosphoramidite method. Several types of non-nucleoside phosphoramidites have been developed for this purpose. The important point is that carborane-modified non-nucleoside units and nucleotides are well compatible, giving mixed-type oligomers (Kane et al., 1993a; Kane et al., 1996) with controllable loading by boron clusters.

### Post-Synthetic Approaches

Two approaches have been proposed for the post-synthetic incorporation of boron clusters into oligonucleotides (Figure 1C). The first one includes the attachment of amino-modified *closo*-dodecaborate to the 5′-terminus of the polymer-bound protected oligonucleotide through the 5′-hydroxyl activation by the disuccinimidyl carbonate (DSC) (Meschaninova et al., 2019). The second approach employs the reaction of azido modified boron clusters with oligonucleotides containing alkyne groups in heterocyclic bases, 2′-positions of ribose moieties, or at 5′-/3′-termini. Its versatility was demonstrated by the syntheses of azido derivatives of electroneutral 1,2-dicarbacloso-dodecaborane, or negatively charged *closo*-dodecaborate and [(3,3′-iron-1,2,1′,2′-dicarbollide) (-1)]ate. The method allows for incorporating one to five clusters per oligonucleotide with good yields (Ebenryter-Olbińska et al., 2017; Kaniowski et al., 2017; Novopashina et al., 2020). While incorporating boron clusters *via* modifications of heterocyclic bases or 2′-ribose has been commonly applied for the synthesis of oligodeoxyribonucleotide conjugates, only one paper describes so far the synthesis of 3′-/5′- terminal conjugates of oligoribonucleotides and their 2′-O-methyl and 2′-fluoro modified analogs (Novopashina et al., 2020).

In general, the post-synthetic incorporation of boron clusters looks more attractive than pre-synthetic approaches due to less excess of boron derivatives required for conjugation and the absence of contact of boron clusters with amines. The latter can convert *closo*-carborane to *nido*-carborane, causing the loss of boron atom and emergence of chirality.

## Properties of functional NA and NA-like oligomeric phosphate diesters bearing boron clusters

So far, several types of functional nucleic acids bearing boron clusters in different positions (Figure 2) were reported, such as antisense oligodeoxyribonucleotides (Ebenryter-Olbińska et al., 2017; Kaniowski et al., 2017; Kaniowski et al., 2020a; Kaniowski et al., 2020b) and siRNA (Kwiatkowska et al., 2013).

These new types of modification influence the key physico-chemical and biological characteristics of the functional nucleic acids, which are described in some detailed reviews (Lesnikowski et al., 1999; Lesnikowski, 2003; Lesnikowski et al., 2004; Olejniczak et al., 2018). In particular, the introduction of boron clusters into oligonucleotides may influence their lipophilicity and capability to cell penetration, nuclease resistance, as well as the thermal stability of their duplexes with complementary DNA and RNA, and characteristics of the double helix. Such effects depend on the nature of boron clusters. Interestingly, the *nido*-form of *ortho*-carboranyl residue exists as two diastereomers and provides the formation of diastereomers of carborane-modified oligonucleotides. They can be used independently, giving various biological effects due to the difference in their properties (Lesnikowski et al., 2004). The influence of the boron cluster on duplex structures is minimal when introduced into 2′-positions, heterocyclic base, or at 3′-terminus of oligonucleotide (Kwiatkowska et al., 2013; Ebenryter-Olbińska et al., 2017; Kaniowski et al., 2017). Meanwhile, the simultaneous modification of 3′- and 5′-termini of DNA oligonucleotide causes a prominent structural rearrangement of complementary complexes with a substantial shift from the B-form to the A-form of the double helix (Novopashina et al., 2020).

Functional nucleic acids supplied with boron clusters represent a dual-action therapeutic platform, acting both as potential ^10^B carriers for BNCT and as gene expression inhibitors. The biological properties of these modified functional nucleic acids are especially interesting in the light of biological and medical applications, especially for BNCT.

The example of boron-modified siRNA duplexes targeting beta-secretase (BACE1) mRNA showed that incorporating N3-carboranyl-modified thymidine into their 3′- and 5′-end strategic positions affects siRNA properties (Kwiatkowska et al., 2013). Such modified siRNA combine the advantages of improved nuclease resistance, the same silencing potential as for non-modified siRNA, and low cytotoxicity, which may be beneficial for biological applications.

*In vitro* studies of antisense oligodeoxynucleotides bearing boron clusters were also reported, such as inhibition of the epidermal growth factor receptor (EGFR) gene expression (Ebenryter-Olbińska et al., 2017; Kaniowski et al., 2017; Kaniowski et al., 2020a). Antisense oligonucleotides with one boron cluster in the middle of a sequence demonstrated improved antisense action (Ebenryter-Olbińska et al., 2017). Among such conjugates, oligonucleotides bearing negatively charged *closo*-dodecaborate showed better effect than those with neutral carboranes. The attachment of several boron clusters to the 2′-positions of ribonucleoside inserts within antisense DNA significantly destabilized their complementary complexes with DNA and RNA. Thereby, higher concentrations of these modified oligonucleotides are required to achieve the antisense effect compared to parent non-modified analogs (Kaniowski et al., 2017). Interestingly, oligonucleotide conjugates bearing several boron clusters can either increase or decrease the amount of reactive oxygen species inside the cells, depending on the conjugate concentration (Kaniowski et al., 2017).

On the contrary to the nuclease protective effect of 5′-carborane modifications, the incorporation of metallacarborane at the 5′-end or at the 3′-end of antisense DNA (Olejniczak et al., 2003; Olejniczak, 2011; Olejniczak et al., 2013; Kaniowski et al., 2020b) unexpectedly promoted its hydrolysis by snake venom phosphodiesterase (svPDE, 3′→5′-exonuclease), probably due to enhanced affinity of metallacarborane moiety to the proteins (Kaniowski et al., 2020b). The original ring-shape nanoconstructs comprising antisense oligonucleotides and boron clusters-modified DNA (Kaniowski et al., 2020a) demonstrated their possible applications as vectors for functional nucleic acids.

Oligomeric phosphate diesters containing carboranes are quite similar to nucleic acids, non-cytotoxic, provide high boron loading (up to 400 atoms), and good water solubility thanks to anionic phosphate backbone (Lesnikowski et al., 2004). Oligomeric phosphate diesters containing *nido*-carborane cages form a mixture of diastereomers due to the chirality of the *nido*-carborane cluster, which can influence some of their physical, chemical, and biological properties. *Nido*-carborane-bearing oligomeric phosphate diesters accumulate in the nucleus after microinjection into the cytoplasm (Nakanishi et al., 1999). Together with excellent compatibility with oligonucleotide synthesis, these characteristics make them especially promising for future BNCT applications.

So far, none of the oligonucleotide or NA-like oligomeric phosphate diester conjugates with boron clusters has been evaluated as candidate BNCT agents, despite their apparent advantages for this purpose. In our opinion, the lack of readily available epithermal neutrons sources necessary for the investigation of these constructs represents one of the main obstacles to their future development.

## Future Directions

Modified functional nucleic acids (antisense/antigene oligonucleotides, siRNA, etc.) (e.g., Wickstrom, 2015; Nuzzo et al., 2019; Roberts et al., 2020) represent a perspective platform for the design and investigations of new high ^10^B-loaded candidate BNCT agents. The use of properly optimized combinations of NA chemical modifications and conjugation to cell/tissue targeting ligands or nanoparticle carriers can enhance the efficacy and selectivity of boron-loaded multimodal NA-constructions. These benefits are further supplied by minimal toxicity and immunogenicity (see, e.g., Laurentia and Rodica, 2016; Bamburowicz-Klimkowska et al., 2019; Hu et al., 2020 and references therein).

Moreover, from our point of view, the very attractive approach to the introduction of multiple boron clusters into oligonucleotides is represented by conjugation with NA-like boron-rich oligomeric phosphate diesters ("boron trailers") due to the possibility to deliver an increased amount of ^10^B atoms into the cells for BNCT cancer therapy (see references in *Solid-Phase Approaches* and *Properties of functional NAs and NA-like oligomeric phosphate diesters bearing boron clusters*).

The considerable scientific experience accumulated in the field of design of boron-modified oligonucleotide constructs paves the way to the new and higher level of their applications. This task calls for a systematic examination of the therapeutic potential of high boron-loaded nucleic acid constructs in the BNCT context and novel interdisciplinary studies using modern sources of epithermal neutrons.
